# Coexistence of carcinoid tumor and adenocarcinoma of the lung; morphological, immunohistochemical and genetic analyses, a case report

**DOI:** 10.1186/s13000-022-01208-5

**Published:** 2022-02-10

**Authors:** Chihiro Inoue, Sachiko Konosu-Fukaya, Kazuhiro Murakami, Ryoko Saito-Koyama, Hirofumi Watanabe, Hideki Mitomo, Naoya Ishibashi, Takafumi Sugawara, Toshiharu Tabata, Hironobu Sasano, Yasuhiro Nakamura

**Affiliations:** 1 Department of Anatomic Pathology, Tohoku University School of Medicine, 2-1 Seiryo-machi, Aoba-ku, Sendai, Miyagi J 980-8575 Japan; 2 Personalized Medical Center, Tohoku University Hospital, Sendai, Miyagi Japan; 3 Division of Pathology, Tohoku Medical and Pharmaceutical University, Sendai, Miyagi Japan; 4 Department of Pathology, Tohoku University Hospital, Sendai, Miyagi Japan; 5 Department of Thoracic Surgery, Tohoku Medical and Pharmaceutical University, Sendai, Miyagi Japan

**Keywords:** Lung cancer, Adenocarcinoma, Carcinoid tumor, Collision tumor, Composite tumor, Case report

## Abstract

**Background:**

Pulmonary carcinoid tumors rarely coexist with non-small cell lung carcinoma, and only nine cases have been reported previously. The pathogenesis and origin of these combined tumors remain unclear because of its rarity.

**Case presentation:**

We examined two cases of adenocarcinoma coexisting with a typical or atypical carcinoid tumor: Case 1 was a 77-year-old woman and Case 2 was an 83-year-old woman. Both of these cases had no respiratory symptoms, and underwent pulmonary lobectomies due to incidentally detected lung nodules. Recurrence and metastases were not detected after the surgery. Histologically, carcinoid and adenocarcinoma components were present in both cases. The two components coexisted without mixing with each other. Next-generation sequencing was performed on the two components in these cases. In each case, no common genetic variants were detected.

**Conclusion:**

We considered that our cases could histologically and genetically represent collision tumors that did not share common progenitor cells. Comprehensive analyses such as whole genome sequencing could provide important information for elucidating the pathogenesis of adenocarcinoma and carcinoid components.

## Background

Neuroendocrine (NE) lung tumors are classified into typical and atypical carcinoid tumors, large cell neuroendocrine carcinoma (LCNEC), and small cell lung carcinoma (SCLC). Carcinoid tumors are rare lung tumors, accounting for < 1% of all malignant lung tumors. A typical carcinoid tumor is a low-grade NE neoplasm with < 2 mitoses/2 mm^2^ without necrosis. Meanwhile, an atypical carcinoid is an intermediate-grade malignancy of 2–10 mitoses/2 mm^2^ with or without necrosis [1]. Carcinoid tumors rarely coexist with non-small cell lung carcinoma (NSCLC). Based on pre-existing literature, only nine cases have [2–9]. Therefore, the pathogenesis and origin of these combined carcinoid tumors remain virtually unknown. We report two cases of adenocarcinoma histologically coexisting with carcinoid tumors and discuss tumorigenesis based on the findings, including the results of genetic analyses in conjunction with a literature review.

## Case presentation

### Case 1

**Fig. 1:**
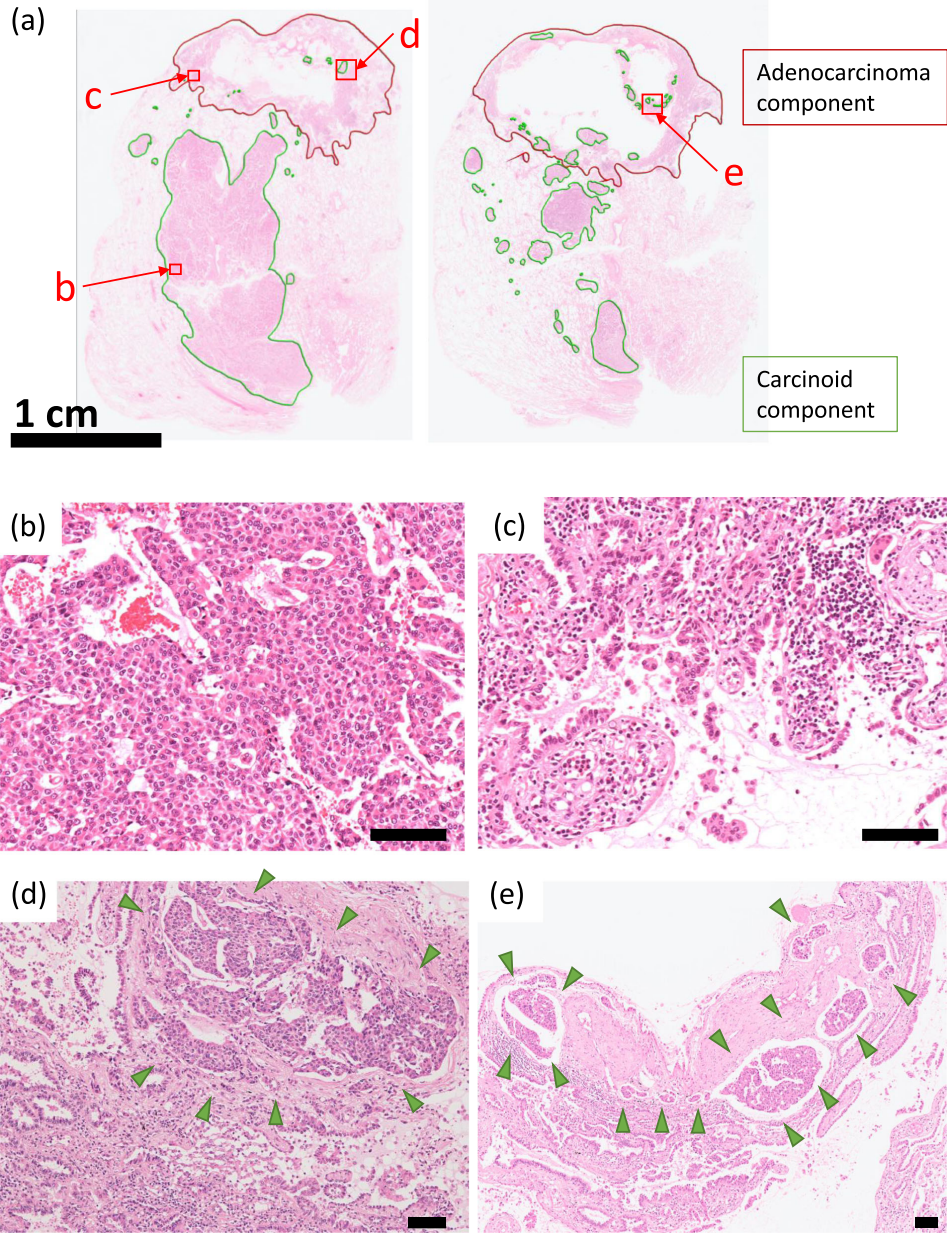
**A** Loupe images of two sections contain a solid lesion (atypical carcinoid: green line) and a cystic lesion (papillary adenocarcinoma: red line), which coexisted in proximity. The atypical carcinoid infiltrated into the papillary adenocarcinoma. **B** The atypical carcinoid, having finely granular nuclear chromatin, proliferated with an organoid pattern. **C** The wall of the cystic lesion was papillary adenocarcinoma. **D, E** The atypical carcinoid spread through veins and lymphatic vessels, and nests of carcinoid tumor (arrowhead) were observed in the adenocarcinoma component. There was a clear demarcation between the two tumor parts, without any histological transitions or intermingling between the two types of tumor cells. [**b–e**: bar = 100 μm]

A 77-year-old woman with a history of parathyroid adenocarcinoma underwent left upper lung lobectomy due to a mass detected during a routine computed tomography scan. She had no history of smoking. Recurrence and metastases were not detected after the surgery. Macroscopically, the mass appeared white on the cut surface, measuring 29 mm in the greatest dimension. A cystic lesion was detected close to the mass. Microscopically, the mass and cystic lesions had different histology (Fig. 1a). The mass was an atypical carcinoid (24-mm in diameter) with an organoid pattern and granular nuclear chromatin without necrosis (Fig. 1b). It showed lymphatic invasion, vascular invasion, and 5 mitoses/2 mm^2^. Immunohistochemically, the tumor cells were positive for CAM5.2, CD56, NSE, chromogranin A, synaptophysin, insulinoma-associated protein 1 (INSM1), and thyroid transcription factor-1 (TTF-1) clone SPT24. It was negative for 34βE12, TTF-1 clone 8G7G3/1, napsin A, surfactant protein A (SP-A), carcinoembryonic antigen (CEA), and p53. The Ki-67 labeling index (LI) was 16.7% at the hot spot (Fig. 3, Table 1). The cystic lesion was a 17-mm papillary adenocarcinoma (Fig. 1c). Immunohistochemically, adenocarcinoma cells were positive for 34βE12, CAM5.2, TTF-1 clone SPT24 and 8G7G3/1, napsin A, SP-A, and CEA. The cells were negative for p53, CD56, NSE, chromogranin A, synaptophysin, and INSM1. The Ki-67 LI was < 1% (Fig. 3, Table 1). The carcinoid tumor infiltrated the adenocarcinoma component through the veins and the lymphatic vessels (Fig. 1a, e). There was a clear demarcation between the two types of tumor cells, without any histological transitions or intermingling between the two types of tumor cells (Fig. 1d, e). Other abnormalities, such as emphysema, fibrosis, or pneumonia, were not observed in the background lung.

**Table 1:** Results of immunohistochemistry

Antibody	Case 1 Ad	Case 1 NE	Case 2 Ad	Case 2 NE
34βE12	+	–	+	–
CAM5.2	+	+	+	+
TTF-1 (clone: SPT24)	+	+	+	+
TTF-1 (clone: 8G7G3/1)	+	–	+	–
CEA	+	–	+	–
Napsin A	+	–	+	–
SP-A	+	–	+	–
CD56	–	+	–	+
NSE	–	+	–	+
chromogranin A	–	+	–	+
synaptophysin	–	+	–	+
INSM1	–	+	–	+
p53	–	–	–	–
Ki-67 LI	< 1%	16.7%	2%	3.2%

NOTE *Ad* adenocarcinoma component, *NE* neuroendocrine tumor component, *LI* labeling index

### Case 2

**Fig. 2:**
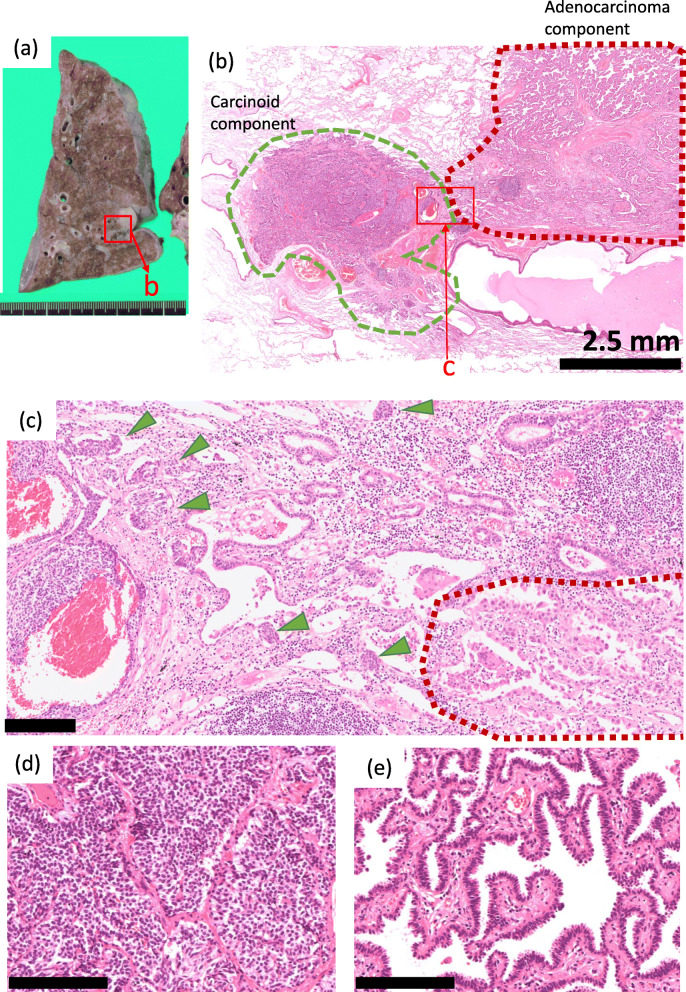
**A** A macroscopic image of the cut lung surface. A white mass with pleural indentation was observed on the cut surface of the lung. **B** A loupe image of the mass. A nodule of carcinoid (green line) was observed close to the adenocarcinoma (red line). **C** A middle-power view of the boundary area of carcinoid (arrowhead) and adenocarcinoma (red line). The two components were distinct. Mild inflammation and fibrosis were observed between the two components. **D, E** High-power views of the carcinoid (**d**) and adenocarcinoma (**e**). [**c–e**: bar = 200 μm]

**Fig. 3:**
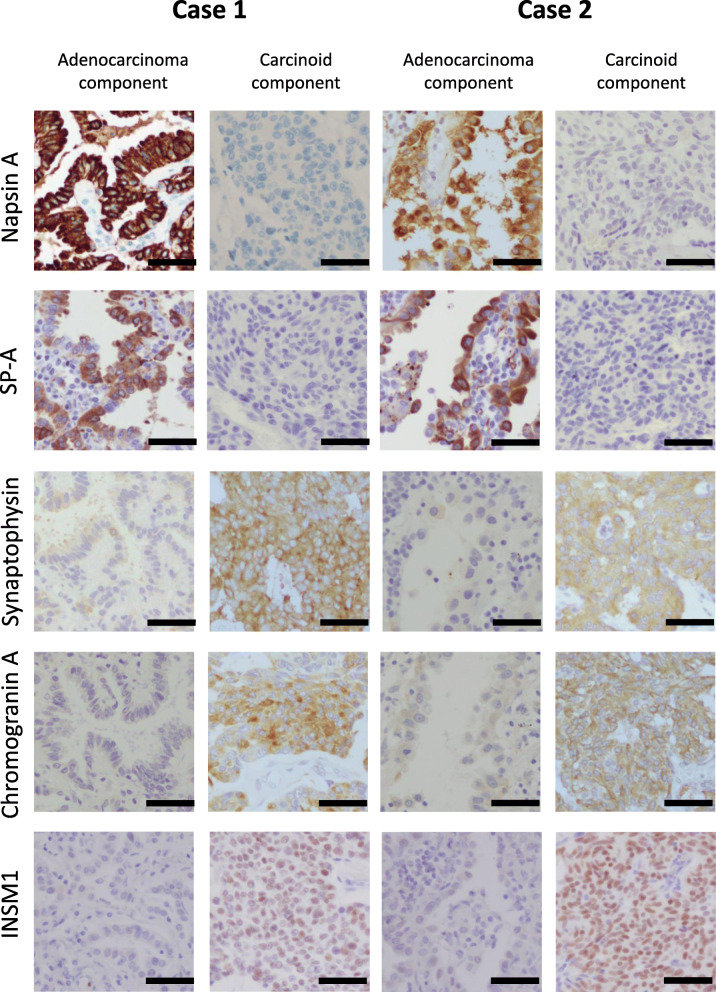
Examples of immunohistochemistry of napsin A, surfactant protein A (SP-A), synaptophysin, chromogranin A, and insulinoma-associated protein 1 (INSM1). Adenocarcinoma components in case 1 and 2 were positive for napsin A and SP-A, and negative for synaptophysin, chromogranin A, and INSM1. Carcinoid components in case 1 and 2 were positive for synaptophysin, chromogranin A, and INSM1, and negative for napsin A and SP-A. Bar = 50 μm

An 83-year-old woman with a smoking history of five cigarettes/day for 3 years also underwent left upper lung lobectomy due to a mass detected via chest radiography. Recurrences and metastases were not detected after surgery. Macroscopically, the mass appeared white on the cut surface, measuring 25 mm in the greatest dimension (Fig. 2a). Microscopically, two different tumors, a typical carcinoid with 1 mitosis/2 mm^2^ (6 mm) and papillary adenocarcinoma (24 mm), were detected (Fig. 2b, c). The carcinoid component was composed of atypical cells with granular nuclear chromatin. It showed an organoid pattern in the middle of the tumor (Fig. 2d). A smaller cluster of atypical cells was observed in the peripheral area (Fig. 2c). The adenocarcinoma component had a papillary pattern (Fig. 2e). A lepidic pattern was observed in the peripheral area, adjacent to the carcinoid component. Immunohistochemically, carcinoid cells were positive for TTF-1 clone SPT24, CAM 5.2, 34βE12, NSE, CD56, chromogranin A, synaptophysin and INSM1. The cells were negative for CEA, TTF-1 clone 8G7G3/1, napsin A, SP-A, and p53. Ki-67 LI was 2%. Adenocarcinoma cells were positive for 34βE12, CAM5.2, TTF-1 clone SPT24 and 8G7G3/1, CEA, napsin A, and SP-A while negative for p53, NSE, CD56, chromogranin A, synaptophysin and INSM1. The Ki-67 LI was 3.2% (Fig. 3, Table 1). There was a clear demarcation detected between these two parts of the lesion. Fibrosis and moderate infiltration of lymphocytes were also detected at the junctional regions of these two parts. Chromogranin A-negative ciliated columnar epithelia were identified at their boundary (Fig. 2c). Focal and very mild infiltrations of lymphocytes were observed in the pleura and bronchioles of the background lung, but they were considered as non-specific changes.

### Genetic analysis

We then examined the mutational status of the two tumor components which are adenocarcinoma and carcinoid tumor. Each tumor component was microdissected from the unstained slides for DNA extraction. Next-generation sequencing was performed using TruSight Tumor 15 (Illumina Inc., San Diego, USA); a targeted cancer gene panel was used for detecting variants by covering the implicated coding regions of 15 genes (*AKT1*, *BRAF, EGFR, ERBB2, FOXL2, GNA11, GNAQ, KIT, KRAS, MET, NRAS, PDGFRA, PIK3CA, RET,* and *TP53*) associated with solid tumors. The adenocarcinoma component of case 2 harbored the *EGFR* L858R mutation, whereas no mutations were detected in other components. Both components of cases 1 and 2 were positive for MLH1, MSH2, PMS2, and MSH6, which are microsatellite stable patterns.

## Discussion and conclusions

Here, we report two rare cases of carcinoid tumors coexisting with adenocarcinomas. The carcinoid data, combined with previously reported NSCLCs, are summarized in Table 2. Although most patients had a smoking history, one of the two cases examined had no history of smoking. Cigarette smoking is considered a risk factor for multiple primary tumors [10]. We examined the expression of common mismatch repair proteins by immunohistochemistry, but a microsatellite instability pattern, which causes multiple primary cancers, was not observed in both cases. Further studies are required to determine the relationship between smoking and the pathogenesis of collision tumors formed by carcinoid tumor and NSCLC in the future. Ki-67 LI is expected to help classify NE tumors of the lung [1]. However, only one of the previous reports assessed Ki-67 LI. Owens et al. [3] reported Ki-67 LI values of < 1 and < 50% for typical carcinoid and squamous cell carcinomas, respectively. In our cases, carcinoid and adenocarcinoma located in the same nodule showed different Ki-67 LI values. In case 1, the atypical carcinoid component aggressively invaded the adenocarcinoma component. This rare coexistence of carcinoid tumors and NSCLC maybe due to tendency of the more aggressive component with higher Ki-67 LI to invade and overwhelm the other components and cause them to disappear. Further experiments using animal models are required to confirm this hypothesis.

**Table 2:** Previous reports of patients with non-neuroendocrine tumor + typical or atypical carcinoid in the lung

	Histology	Age/Sex	Smoking	Location	Ki-67 LI in TC/AC	Ki-67 LI in Ad/Sq	Tumor size	Metastasis/Histology	Reccurence	Survival/death	Refference
1	TC + Ad	60/M	+	L/Peripheral	NA	NA	20 mm	Lymph node/Ad	–	Survival	Sen, et al. 1998 [2]
2	TC + Sq	71/F	+	L/Central	< 1%	< 50%	50 mm	Lymph node/NA	NA	NA	Owens, et al. 2011 [3]
3	TC + Ad	67/F	+	R/Peripheral	NA	NA	18 mm	–	–	Survival	Nagamatsu, et al. 2011 [7]
4	TC + Ad	74/F	+	L/Peripheral	NA	NA	13 mm	–	NA	NA	Abbi, et al. 2014 [4]
5	AC + Sq	71/M	+	R/Peripheral	NA	NA	70 mm	–	Metastases to brain and adrenal	Died of pneumonia, 21 months after the surgery	Okazaki, et al. 2015 [5]
6	AC + Ad	66/M	+	R/Peripheral	NA	NA	17 mm	–	–	Survival	Olofson, et al. 2018 [6]
7	AC + Ad	NA	NA	Peripheral	NA	NA	NA	NA	–	Survival	Ruffini, et al. 2002 [8]
8	TC + Sq	NA	NA	Peripheral	NA	NA	NA	NA	–	Survival	Ruffini, et al. 2002 [8]
9	Carcinoid, NOS + Ad	53/M	NA	R	NA	NA	NA	NA	NA	NA	Li, et al. 2015 [9]
10: Case 1	AC + Ad	77/F	-	L/Peripheral	16.7%	< 1%	29 mm	–	–	Survival	Present case
11: Case 2	TC + Ad	83/F	+	L/Peripheral	2.0%	3.2%	25 mm	–	–	Survival	Present case

NOTE: *TC* Typical carcinoid, *AC* Atypical carcinoid, *Ad* Adenocarcinoma, *Sq* Squamous cell carcinoma, *NOS* not otherwise specified,

*M* Male, *F* Female, *L* Left, *R* Right, *NA* Not available, *Ki-67 LI* Ki-67 labeling index

In general, driver mutations common in NSCLC are rare in pulmonary carcinoid tumors [6, 11]. Pulmonary carcinoid tumors are derived from pulmonary NE cells (PNECs) [11]. In contrast, primary lung adenocarcinoma originates from club and alveolar type II (ATII) cells, which are derived from bronchioalveolar stem cells [11]. Therefore, the pathogenesis of pulmonary carcinoid tumors could be different from that of NSCLC. However, Olofson et al. reported a case of a tumor with adenocarcinoma and atypical carcinoid harboring *BRAF* p.V600E, a rare mutation in these tumors. They hypothesized that these components might be derived from a shared progenitor cell with a *BRAF* mutation [6]. Their hypothesis is plausible because both PNECs and bronchioalveolar stem cells are derived from epithelial precursor cells [11]. However, common gene mutations and abnormal expression of p53 in the two components were not detected in the mutational statuses of adenocarcinoma and carcinoid components in our cases. In addition, *EGFR* mutations in case 2 were detected only in the adenocarcinoma component. Therefore, two hypotheses regarding the pathogenesis of case 2 can be proposed: (1) both carcinoid tumor and adenocarcinoma cells were derived from the same epithelial precursor cells, and *EGFR* mutations occurred after differentiation into bronchioalveolar-type cells; (2) adenocarcinoma and carcinoid cells have different precursor cells and grew into synchronous collision tumors. Coexisting tumors are generally divided into two types: composite and collision tumors [4, 12]. Composite tumors are derived from the same progenitor cell, and therefore, the two components share the same mutational status. Meanwhile, collision tumors originate from different progenitor cells. Thus, the two components show different mutational status [12]. Histologically, in composite tumors, two components are intermingled and have interposed transitional tumor cells with characteristics of the two components [4]. In our cases, each nodule of the adenocarcinoma and carcinoid components was an individual nodule adjacent to each other. No transitional cells or intermingled patterns were detected morphologically between the two components. In immunohistochemical analysis, each of the two compartments showed a typical immunohistochemical pattern. Adenocarcinoma was positive for SP-A and napsin A, and carcinoid was positive for NE markers, including NSE, CD56, chromogranin A, and synaptophysin. Both components were positive for TTF-1 clone SPT24, whereas only adenocarcinoma components were positive for TTF-1 clone 8G7G3/1. TTF-1 is generally expressed by club and ATII cells in normal lung tissue. However, NE cells are occasionally positive for the TTF-1 SPT24 antibody [13]. Some primary pulmonary carcinoid tumors, especially those located in the peripheral lung, are positive for TTF-1 [14]. Therefore, the immunopositivity for TTF-1 does not indicate a transitional or intermingling pattern of adenocarcinoma and carcinoid. These histological features were consistent with the results of genetic analysis. Therefore, our cases were considered as collision tumors. In the gastroenteropancreatic system, the pathogenesis of NE tumors is associated with hyperplastic changes in NE cells. Ouadah et al. demonstrated that a subpopulation of NE cells in the bronchioles functioned as stem cells [15]. In addition, Reynolds et al. reported that neuroepithelial bodies in bronchioles were a reservoir of progenitor cells and demonstrated features of both club cells and pulmonary NE cells [16]. Some carcinoid tumors are considered to be derived from NE cells via diffuse idiopathic pulmonary NE cell hyperplasia and/or tumorlets [1, 11]. Therefore, these findings could also indicate another hypothesis that paracrine signaling from adenocarcinoma could influence the pathogenesis of NE cell hyperplasia by altering the microenvironment, and subsequently, its tumorigenesis. However, it could not explain why a composite and collision tumor of adenocarcinoma and carcinoid tumors in the lung is extremely rare, compared to the gastrointestinal tract or pancreas. In case 1, no apparent alterations in the tissue microenvironment were detected between the adenocarcinoma and carcinoid components. In case 2, alterations in the microenvironment, such as infiltration of lymphocytes and fibrosis, were detected between these two components, whereas NE cell hyperplasia did not. No NE cell hyperplasia or tumorlets were observed in the background lung tissue in either case. This hypothesis of carcinoid tumors derived from NE cell hyperplasia caused by adenocarcinoma could not explain the origin of the collision tumors in our cases. Therefore, comprehensive studies of the whole-genome sequence analysis of adenocarcinoma and carcinoid are required to elucidate the detailed pathogenesis of collision tumors composed of both adenocarcinoma and NE tumors in the lung.

Here, we report two cases of adenocarcinoma coexisting with carcinoid tumors in the lungs. These cases, histologically and genetically, appeared to be collision tumors originating from each progenitor cell. Further studies based on both histological observations and genetic analyses are required to understand the pathogenesis of these components.

## Data Availability

All data generated or analyzed during this study are included in this published article.

## Abbreviations

NE: Neuroendocrine
LCNEC: large cell neuroendocrine carcinoma
SCLC: Small cell lung carcinoma
NSCLC: non-small-cell lung carcinoma
TTF-1: thyroid transcription factor-1
INSM1: insulinoma-associated protein 1
SP-A: surfactant protein A
CEA: carcinoembryonic antigen
LI: labeling index
PNECs: pulmonary neuroendocrine cells
ATII cells: alveolar type II cells
